# Effect of Oily Sludge Treatment with Molten Blast Furnace Slag on the Mineral Phase Reconstruction of Water-Quenched Slag Properties

**DOI:** 10.3390/ma14237285

**Published:** 2021-11-28

**Authors:** Yuelin Qin, Ke Zhang, Xinlong Wu, Qingfeng Ling, Jinglan Hu, Xin Li, Hao Liu

**Affiliations:** 1 School of Metallurgy and Materials Engineering, Chongqing University of Science and Technology, Chongqing 401331, China; zhangke980710@163.com (K.Z.); wu15234024789@163.com (X.W.); lingqingfeng9762@163.com (Q.L.); hujinglan_cqust@163.com (J.H.); lixin2020202034@163.com (X.L.); liuhao@cqust.edu.cn (H.L.); 2 Value-Added Process and Clean Extraction of Complex Metal Mineral Resources, Chongqing Municipal Key Laboratory of Institutions of Higher Education, Chongqing 401331, China

**Keywords:** blast furnace slag, oily sludge, pyrolysis and digestion, heavy metals

## Abstract

Blast furnace slag, which is the main by-product of the ironmaking process discharged at 1450 °C, contains high-quality sensible heat, while oily sludge is the main solid waste produced in the process of gas exploration, storage, and transportation. The energy and resource utilization of blast furnace slag is complementary to the environmentally friendly treatment of oily sludge, which has provided a new idea for the multi-factor synergistic cycle and energy transformation of the two wastes. The pyrolysis of the oily sludge with the molten blast furnace slag was conducted in the current paper. Results showed that the oily sludge was rapidly pyrolyzed, and the heavy metal elements in the oily sludge were solidified. The solidification rate of the heavy metals exceeds 90%, except for vanadium. The reconstituted water-quenched blast furnace slag still has good activity, and it will not affect the further use of the slag after pyrolysis (BFS-P).

## 1. Introduction

Blast furnace slag (BFS) is the most important by-product in iron and steel production, with an annual emission of more than 300 million tons at a temperature of 1450 °C. One ton of BFS contains the heat equivalent to that of coal. Therefore, BFS is a high-quality waste as a heat source [1,2,3]. BFS is a large and cheap material resource can be used as a secondary energy, but it is hard to utilize the heat from BFS due to its low thermal conductivity and slow heat exchange [4,5,6]. At present, water quenching is a popular technique for slag production, which leads to the wastage of considerable high-quality heat [5,6,7]. Therefore, effectively improving the energy efficiency and reducing the resource consumption to recycling slag, and recovering the high-quality sensible heat, is crucial for the metallurgical industry [8,9,10]. 

Oily sludge (OS) is one of the major oily wastes that is associated with petroleum exploitation, transportation, and processing, which is also highly pollutive and hard to process [11]. OS contains numerous petroleum-type organic compounds and heavy metals. Therefore, improper disposal will cause serious pollution to the surrounding environment [12]. Moreover, OS has been listed as a hazardous solid waste industry by the government [13,14,15]. Statistics show that the annual increase in OS in the oil exploitation in China has exceeded 6 million tons. The treatment gap has reached 2.7 million tons per year, and the accumulated OS in recent years has exceeded 10 million tons [16]. However, environmentally friendly treatment and resource utilization of oil-based drilling cuttings is difficult due to the complex composition and the complicated reutilization of OS [17,18,19,20]. The current treatment processes only meet the criterion to be environmentally friendly, but the material resources contained in OS remain unexploited. Therefore, it is important to promote a strategy to coordinate the development of oil and gas exploration and growth, environmental protection, and resource reutilization in the field of oil and gas [21,22]. At present, the treatment of OS is mainly based on “terminal treatment and disposal”. The existing treatment techniques for OS include the heat treatment process, chemically enhanced solid–liquid separation, bioremediation, and solvent extraction [23,24,25,26]. Among these techniques, the pyrolysis technology is widely used since it has the advantages of thorough disposal, lower pollution, significant reduction of volume and capacity, and high energy reutilization.

The pyrolysis of OS and recycling the heat of BFS reflected a good complementarity, which undoubtedly provides a new idea for the multi-factor synergistic cycle and energy conversion of the two wastes. The pyrolysis of OS requires a large amount of heat, while molten BFS is the byproduct of ironmaking at temperatures of about 1450 °C, carrying a substantial amount of high-quality thermal energy. Due to the low thermal conductivity and slow heat exchange, the heat of BFS is hard to directly recover. A new idea is to use the heat of BFS in co-pyrolysis to treat OS, convert the high-temperature sensible heat of BFS into chemical reaction heat, and produce chemical energy which can effectively recover the heat of the blast furnace slag while harmlessly treating the OS. In addition, the OS contains numerous petroleum-type organic compounds and heavy metals, so the pyrolysis process of OS with BFS will not only recover the waste heat of the blast furnace slag, but also obtain some recyclable pyrolysis products from the OS and can harmlessly treat the heavy metals in OS.

In this study, the experiment of the OS treatment via the thermal pyrolysis of BFS was conducted. Since the BFS can be used to produce the basic raw materials of cement, bricks, and other building materials, etc., the microscopic morphology and the phase composition of the BFS after pyrolysis (BFS-P), and the stability of heavy metal in the BFS-P, were also evaluated in this study. The objectives of this work are to investigate the reliability of the pyrolysis of biomass with hot BFS, to harmlessly treat the OS, and the reutilization of the waste heat of the BFS.

## 2. Materials and Methods

### 2.1. Raw Materials

**Figure 1:**
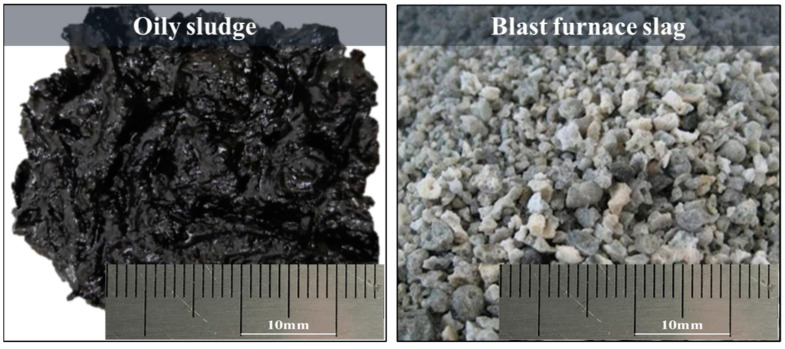
Samples of OS and BFS.

The raw materials used in this experiment include OS provided by No. 1 refining and the chemical plant of Liaohe Oilfield, and the BFS was provided by Chongqing Iron & Steel Co., Ltd. (Chongqing, China). Both are shown in Figure 1. The OS is transported back in rubber drums. Sufficient agitation is conducted before sampling to eliminate the effects of sedimentation and stratification during storage. OS samples are taken from the bottom of the oil-bearing sewage storage tank. The OS contains a large water content at room temperatures. The sludge becomes gray-black and viscous with irritating odor after agitation. The BFS is fine particles dominated by a vitreous body and formed under the condition of rapid cooling. The BFS is fluffy and porous with poor strength, which was caused by the impact of a large amount of water on the BFS at high temperature. The BFS used in the experiment is a typical CaO-SiO_2_-MgO-Al_2_O_3_ quaternary silicate with the binary basicity (CaO/SiO_2_) of 1.2. The chemical composition of the BFS is shown in Table 1. The industrial analysis results of the OS are presented in Table 2. The content of the fixed carbon in the samples is relatively low compared with that of volatile and moisture, while the proportion of volatile content is the largest, followed by moisture and ash. The calorific value of the OS is 28.44 MJ/kg. The heavy metals content in the OS are tested by inductively coupled plasma mass spectrometry (ICP) and the results are shown in Table 3. The heavy metals in the samples mainly include V, Cr, Co, Ni, Cu, Zn, Cd, Sb, and Pb. The content of each heavy metal element varies considerably. Compared with the soil environment quality risk control standard for soil contamination of agriculture land GB 15618-2018, it can be found that the contents of V, Ni, Zn, Sb, and other heavy metals exceeded the maximum allowable emission standards, especially the Zn content, which exceeded by 100 times. 

### 2.2. Experimental Methods

**Figure 2:**
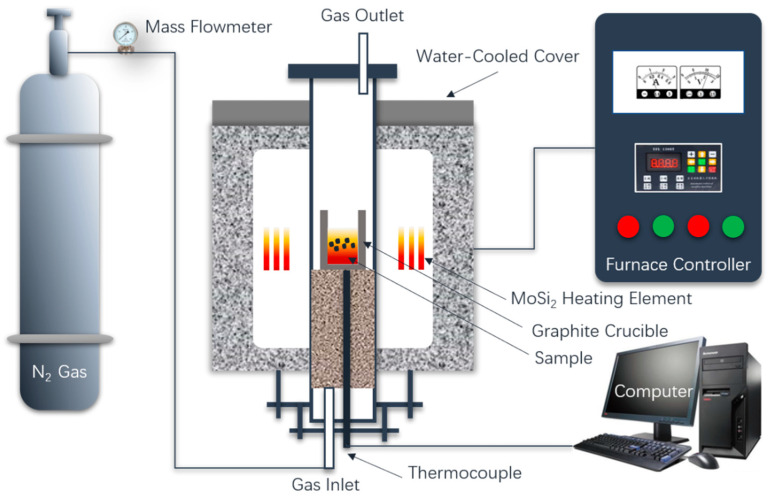
Experimental platform of OS absorption by pyrolysis of BFS.

Figure 2 shows the experimental platform of the pyrolysis of OS by molten BFS. The specific pyrolysis and digestion experiment scheme are shown in Table 4. The target pyrolysis temperature is 1450 °C. For each experiment, the total weight of the BFS and the OS is 100 g, and the blending weight ratio of the BFS and OS is 9:1, 8:2, 7:3, 6:4, and 5:5.

**Figure 3:**
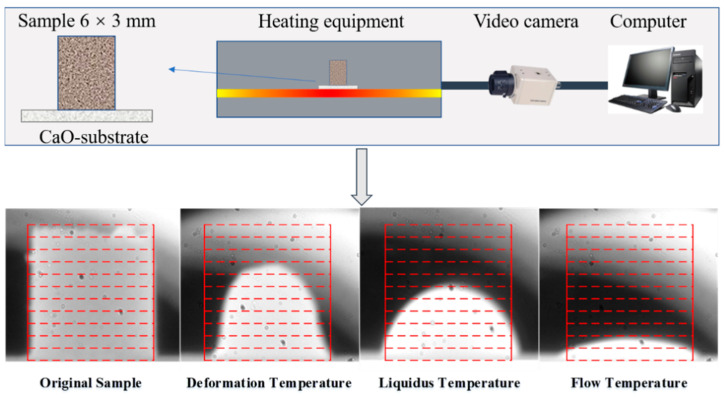
Hemispherical point method.

During the experimental process, the mixture of the original BFS and OS was first charged into a graphite crucible. A high-temperature silicon molybdenum rods furnace covered by a graphite crucible furnace lid is employed to heat the BFS to the target temperature. When the target temperature is reached for the reaction, N_2_ is ventilated into the furnace to control the inert atmosphere, then put the graphite crucible with mixture into the furnace. After holding 15 min at 1450 °C, take out the sample and pour it into the water tank as fast as possible for water quench, followed by filtering. The slag after pyrolysis (BFS-P) was then dried after cooling for 10 min. The BFS-P is used for detection and analysis of the phase structure, the solidification effect of heavy metals, and the melting performance. The melting performance of BFS-P was tested by the hemispherical point method. Figure 3 shows that the BFS-P is made into a column for the melting performance experiment. The starting melting, the hemispherical point, and the complete melting temperatures of the BFS-P were determined based on the height change of the columnar sample. The melting temperature is defined as temperature at which the height of the columnar sample decreased to 70% of the original height. In a similar way, the points at which the heights of the columnar sample decreased to 50% and 20% are defined as the melting point temperature and the full melting temperature, respectively. 

## 3. Results

### 3.1. Analysis of Phase and Morphology of the Slag after Pyrolysis (BFS-P)

**Figure 4:**
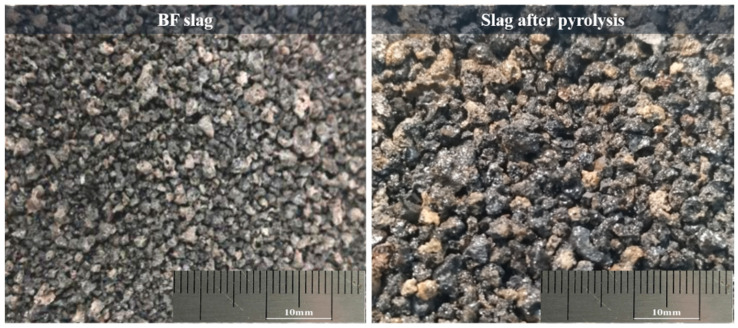
Original BFS and the slag after pyrolysis (BFS-P).

The appearance and morphology of the BFS-P and the original BFS are shown in Figure 4. It can be found that both slags comprising fine glassy particles with a grayish-white surface is rough and porous. Compared with the original BFS, the BFS-P is brittle, has a lower strength, is loose, porous, and fragile. The oily organic matter was cracked as flue gas at high temperature during the process. However, ash and heavy metals from the OS were fixed in the slag to reconstruct the mineral phase of the original BFS.

**Figure 5:**
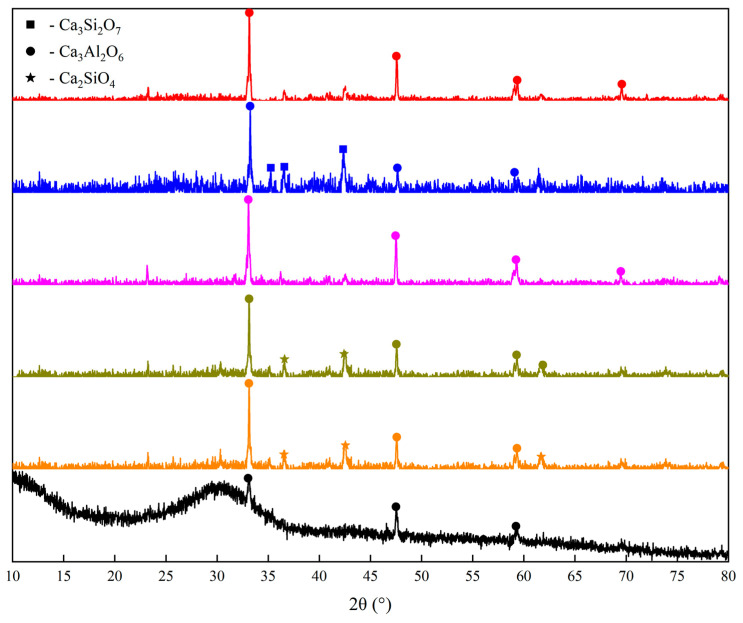
XRD pattern of the slag after pyrolysis (BFS-P) and original BFS.

As can be seen in Figure 5, The main ore phases of the BFS-P significantly changed compared with the original BFS. The original BFS mainly consists of a glass phase with an amorphous peak and substantially small tricalcium aluminate (Ca_3_Al_2_O_6_), while the peak was more obviously in the BFS-P. The main diffraction peak of the BFS is wollastonite (Ca_2_SiO_4_) and Ca_3_Al_2_O_6_. The Ca_3_Al_2_O_6_ diffraction peak in the BFS-P showed a rising trend with an increasing OS mass. The Ca_2_SiO_4_ phase appeared when 10% OS was added into BFS. However, it disappeared with an OS amount higher than 30%, and only Ca_3_Al_2_O_6_ was detected. Ca_3_Si_2_O_7_ appeared in the BFS-P as the amount of OS had a continuous increase. The main crystalline phase of the BFS-P is Ca_3_Al_2_O_6_, when the heat incorporation of the OS is 50%. The results show that the pyrolysis residue of the oil-bearing sludge reconstructed the mineral phase of the original BFS. However, the phase of the BFS-P is consistent with the requirement for cement production. Therefore, the BFS-P can still be used for producing cement and other construction materials according to the results shown in Section 3.2.

**Figure 6:**
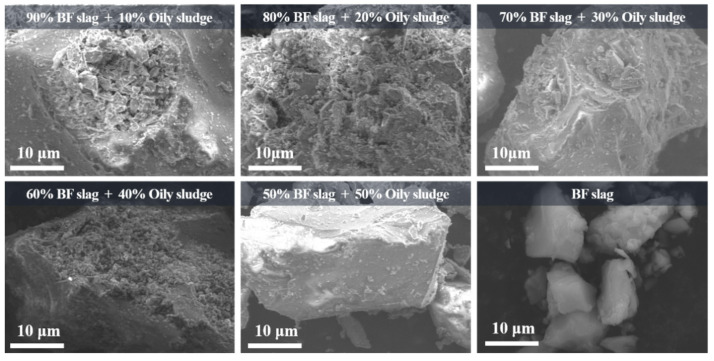
SEM microscopic image of the slag after pyrolysis (BFS-P).

The microscopic morphology of the BFS-P is shown in Figure 6, and a comparative analysis with the original BFS is also provided. It is found that the microscopic morphology of the BFS-P changes to a certain extent with the increase of the OS amount, but the overall change is insignificant. Compared with the original BFS, the BFS-P has a tetragonal morphology, which is short columnar or plate-like, with prominent corners and sharp edges. The particles also have few surface pores. This trend is observed with the increase of the amount of OS, which indicates that the strength of the BFS-P is decreasing.

### 3.2. Analysis of the Solidification Rate and Stability of Heavy Metals in the Reconstruction Slag

The content of heavy metal elements in the OS, BFS, and the BFS-P were analyzed, and are calculated to obtain the solidification rate of heavy metals in the BFS-P. The solidification rate (R_f_) expression is as follows:

(1) $$R_{f}=\left(1-\frac{c_{1}\times m_{0}+m_{2}-c_{2}\times m_{2}}{c_{0}\times m_{0}}\right)\times 100\%,$$

where

c_1_: concentration of heavy metals in the BFS-P, mg/kg;
c_2_: concentration of heavy metals in BFS, mg/kg;
c_0_: concentration of heavy metals in OS, mg/kg;
m_0_: mass of OS, kg;
m_2_: mass of BFS added, kg.

**Figure 7:**
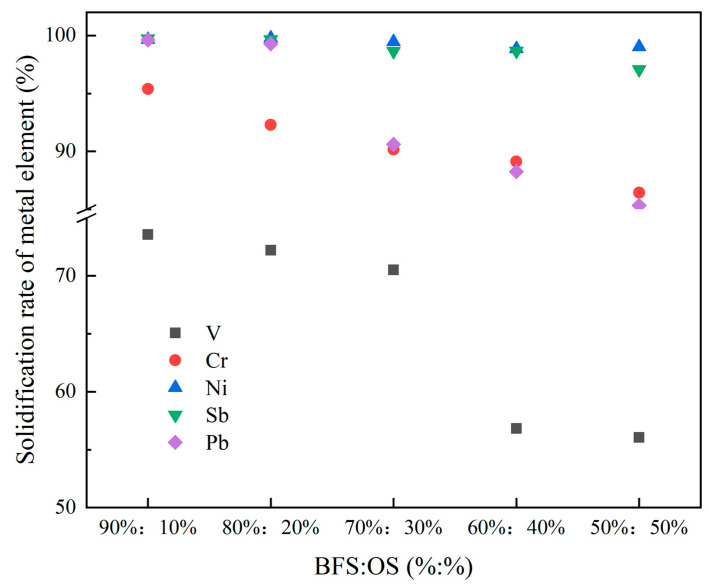
The solidification rate of heavy metal elements.

The solidification rate of the heavy metal elements is shown in Figure 7. It can be found that the solidification rate decreased with the increase of the OS, which indicates that the solidification capability of the slag to the heavy metals is limited. The addition amount of the OS should not exceed 30% of the total weight. The solidification rate of Ni and Pt remain nearly constant and is close to 100% when increasing the OS ratio in the mixture. However, the solidification rate of Sb, Cr, and V significantly decreased as the OS ratio in the mixture increased. Especially for V, the solidification rate decreased from about 74% to 56% when the OS increased from 10% to 50%. It may be caused by the formation of stable phases like VC and V_2_C in slag. Thus, it is an effective way to extract vanadium by using BFS-containing vanadium. Moreover, a relatively complete vanadium extraction process from the molten iron and the BFS has been established, and the BFS-P has a good solidification effect on the V element. The solidification rate of Cr is relatively higher due to its high volatilization point, and the Cr_2_O_3_ formed in the molten BFS with a melting point as high as 2000 °C. However, a certain content of the Pb element is detected only when the content of the OS is 20–30%, and it is undetected in the other series of experiments. Under normal circumstances, the Pb element is volatile at 1450 °C. The Pb element detected in this paper may be encapsulated by silicoaluminate formed by the reaction of Al_2_O_3_, SiO_2_, and other oxides in the molten BFS. Therefore, it becomes stable compounds, and they are retained. Cu and Zn elements were also undetected in the entire experimental series. The analysis shows that Zn does not enter the slag because of its low boiling point and easy volatilization at high temperature. Meanwhile, Cu interacts with organic matter in OS to form CuCl_2_ and other compounds into tail gas. Therefore, Cu is undetected in the BFS-P. 

The leaching of heavy metal elements in the BFS-P was conducted to analyze the stability of the heavy metal elements after solidification. The experimental procedure adopted the “Solid waste leaching toxicity leaching method-sulfuric acid and nitric acid method” (HJ/T 299-2007). Table 5 shows the content of the heavy metals in the leachate. It is found that the leaching amount of V, Cr, Ni, Sb, and Pb is substantially small, which is lower than the value in the “Identification Standard for Hazardous Waste-Identification of Leaching Toxicity” (GB5085.3-2007). The elements of V, Cr, Ni, Sb, and Pb are detected in several leachate, but the V element is far lower than the requirement of “hazardous waste identification standard-leaching toxicity identification (GB5085.3-2007) (3) allowed by the highest concentration of request”, which shows that the solidification effect of the BFS-P for heavy metals is positive compared to the conventional harmless solid waste landfill. The solidification effect reaches the best when the ratio of the BFS to the OS residue is 7:3. In this condition, V, Cr, Ni, and other elements in the leaching solution are undetected, and the leaching amount of Sb is also low. According to the above analysis, it can be found that the structure and composition of the BFS-P do not change substantially. Therefore, the BFS-P can also be used to produce the basic raw materials of cement, bricks, and other building materials, as well as filling and roadbed materials in mining areas and mines, to realize resource reutilization. The results show that the solidification stability of the BFS-P for heavy metals meets the requirement of the basic raw building materials, such as cement and brick, filling materials of mining pits, and roadbed materials.

### 3.3. Reconstruction Slag Melting Performance

**Figure 8:**
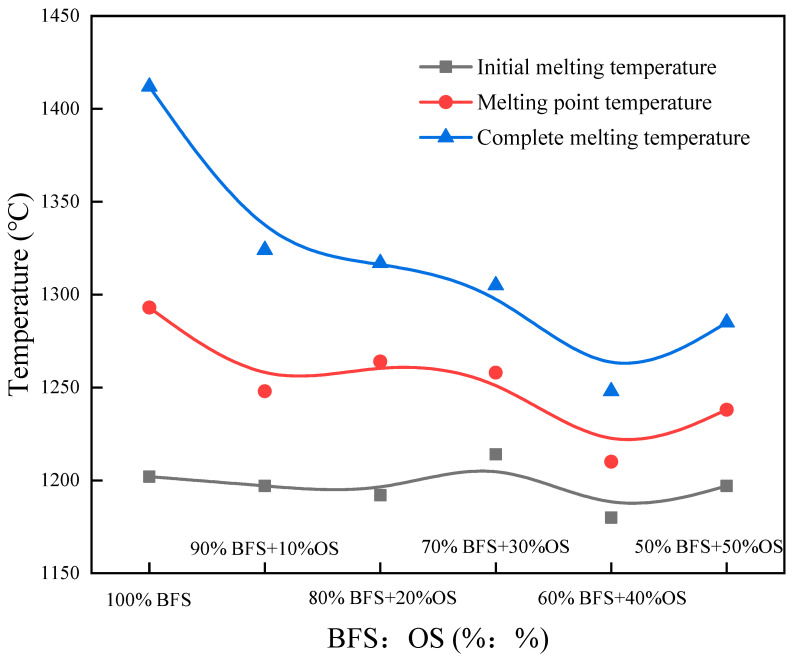
Melting properties of the slag after pyrolysis (BFS-P).

The melting performance of the BFS-P is shown in Figure 8. The increase of OS ratio has slight effect on the melting temperature of the BFS-P, and the melting temperature maintained at approximately 1200 °C. The total melting and melting point temperatures decreased with the increase of the OS ration, and the melting interval of the BFS-P also decreased. The results show that the pyrolysis residue of the oil-bearing sludge can significantly reduce the melting temperature of the original BFS and promote the formation of the liquid phase. Therefore, the slag has improved fluidity at high temperatures.

## 4. Conclusions

(1) The pyrolysis process of the oily sludge with the molten BFS not only effectively treats the sludge and reuses the heat of the BFS, but also solidifies the heavy metal of OS with BFS. The solidification rate decreases with the increase of OS, and the appropriate absorption proportion of the OS is not more than 30%.

(2) The BFS-P has a good solidification stability of heavy metals. The crystallite phase of the BFS-P includes kilchoanite, tricalcium aluminate, and calcium silicate, which can still be used in the production of cement and other building materials.

(3) The melting point of the formed reconstituted slag decreases with the increase of the OS. The melting range shows a shrinking trend, and the BFS-P fluidity is good.

## Figures and Tables

**Table 1:** Chemical composition of BFS samples.

CaO	SiO_2_	Al_2_O_3_	MgO	R(CaO/SiO_2_)
43.64	36.36	11.80	8.05	1.20

**Table 2:** Industrial analysis results of oily sludge.

Proximate Analysis (Weight Content %)				Heat Value (MJ/Kg)
Moisture	Volatiles	Ash	Fixed Carbon	
30.16	40.88	24.47	3.48	28.44

**Table 3:** Heavy metal content of oily sludge.

Metal Element	V	Cr	Ni	Cu	Zn	Cd	Sb	Pb
Oily sludge (mg/kg)	132.5	165.4	225.3	75.7	218.4	12.2	101.4	482.7
GB 15618-2018 (mg/kg)	—	350.0	190.0	200.0	300.0	0.8	40.0	170.0

**Table 4:** Experimental plan for the pyrolysis of OS by BFS.

No.	Mixture Ratio (BFS:OS)	Temperature (°C)	Total Mass of Sample (g)
1	90%:10%	1450	100
2	80%:20%	1450	100
3	70%:30%	1450	100
4	60%:40%	1450	100
5	50%:50%	1450	100

**Table 5:** Leaching results of heavy metals in the slag after pyrolysis (BFS-P).

Sample No.	Leaching Content (mg/kg)						
	V	Cr	Cu	Zn	Ni	Sb	Pb
1	0.04 ± 0.003	0.34 ± 0.005	—	—	3.45 ± 0.007	1.63 ± 0.004	—
2	—	0.02 ± 0.001	—	—	0.80 ± 0.004	6.26 ± 0.002	0.69 ± 0.002
3	—	—	—	—	0.65 ± 0.003	0.95 ± 0.003	1.92 ± 0.007
4	—	—	—	—	0.07 ± 0.005	2.85 ± 0.002	—
5	—	0.37 ± 0.006	—	—	0.04 ± 0.003	2.46 ± 0.003	—
(GB5085.3-2007) (3) (mg/kg)	—	10.00	—	—	10.00	10.00	3.00

## Data Availability

Data are contained within the article.
